# Ancestry and evolution of a secretory pathway serpin

**DOI:** 10.1186/1471-2148-8-250

**Published:** 2008-09-15

**Authors:** Abhishek Kumar, Hermann Ragg

**Affiliations:** 1 Department of Biotechnology, Faculty of Technology and Center for Biotechnology, University of Bielefeld, D-33501 Bielefeld, Germany

## Abstract

**Background:**

The serpin (serine protease inhibitor) superfamily constitutes a class of functionally highly diverse proteins usually encompassing several dozens of paralogs in mammals. Though phylogenetic classification of vertebrate serpins into six groups based on gene organisation is well established, the evolutionary roots beyond the fish/tetrapod split are unresolved. The aim of this study was to elucidate the phylogenetic relationships of serpins involved in surveying the secretory pathway routes against uncontrolled proteolytic activity.

**Results:**

Here, rare genomic characters are used to show that orthologs of neuroserpin, a prominent representative of vertebrate group 3 *serpin *genes, exist in early diverging deuterostomes and probably also in cnidarians, indicating that the origin of a mammalian serpin can be traced back far in the history of eumetazoans. A C-terminal address code assigning association with secretory pathway organelles is present in all neuroserpin orthologs, suggesting that supervision of cellular export/import routes by antiproteolytic serpins is an ancient trait, though subtle functional and compartmental specialisations have developed during their evolution. The results also suggest that massive changes in the exon-intron organisation of *serpin *genes have occurred along the lineage leading to vertebrate *neuroserpin*, in contrast with the immediately adjacent *PDCD10 *gene that is linked to its neighbour at least since divergence of echinoderms. The intron distribution pattern of closely adjacent and co-regulated genes thus may experience quite different fates during evolution of metazoans.

**Conclusion:**

This study demonstrates that the analysis of microsynteny and other rare characters can provide insight into the intricate family history of metazoan serpins. Serpins with the capacity to defend the main cellular export/import routes against uncontrolled endogenous and/or foreign proteolytic activity represent an ancient trait in eukaryotes that has been maintained continuously in metazoans though subtle changes affecting function and subcellular location have evolved. It is shown that the intron distribution pattern of *neuroserpin *gene orthologs has undergone substantial rearrangements during metazoan evolution.

## Background

The serpins represent a superfamily of proteins with a common fold that cover an extraordinary broad spectrum of different biological functions. Most serpins inhibit proteases from one or several different clans of peptidases; some superfamily members, however, exert disparate roles, such as assisting in protein folding or transportation of hormones [1]. This functional diversity is enabled, at least in part, by the unusual structural plasticity of the serpin molecule that, in the native form, often takes a metastable structure. Serpins can perform their activity in the extracellular space or in various subcellular compartments, including the secretory pathway routes [2,3], and they are found in all high-order branches of the tree of life [4]. Deficiency of some serpins, such as antithrombin or neuroserpin, is lethal or may be associated with serious pathology [5,6]. Mutations of the *neuroserpin *gene for instance may result in formation of intracellular aggregates in the brain causing dementia [6], while wild type neuroserpin provides protection of neuronal cells in cerebral ischemia and other pathologies [7]. Neuroserpin inhibits tissue plasminogen activator (tPA), urokinase-type plasminogen activator, nerve growth factor-γ, and plasmin. These enzymes are also believed to represent physiological targets of the inhibitor [8,9]. Native neuroserpin is found in the medium of some cell lines [9] but also in dense core secretory vesicles of neuronal cells [10,11], suggesting that it could exert a function within the regulated secretory pathway, though there is no experimental evidence for this. Association of neuroserpin with secretory pathway organelles is mediated via a 13 amino acid C-terminal sorting sequence [11]. Recently, serpins equipped with a C-terminal endoplasmic reticulum (ER) retention/retrieval signal that efficiently inhibit furin and/or other members of the proprotein convertase (PC) family have been identified in *Drosophila melanogaster *[12-15], demonstrating for the first time that serpins with antiproteolytic activity may reside in early secretory pathway organelles.

The elucidation of phylogenetic relationships among animal serpins poses a notorious problem [16]. *Serpin *genes represent a substantial fraction of metazoan genomes, often amounting to several dozens of members in mammals. In various vertebrate lineages multiple expansions of *serpin *genes have occurred [17,18] resulting in numerous paralogs. In other lineages, such as fungi, serpins seem to be rare. In some species phylogenetic relationships of *serpin *genes may be obscured further by a propensity for reciprocal or non-reciprocal exchange of cassette exons coding for the hypervariable reactive site loop region (RSL) [19]. The sequence of this region plays a primary role in determining the specificity of serpin/target enzyme interaction. Inhibition of target proteases involves cleavage of a scissile bond located between positions P1 and P1' of the inhibitor's RSL [1]. Serpins also occur with a patchy distribution in prokaryotes, but the time point of their first emergence is not known [4].

**Figure 1:**
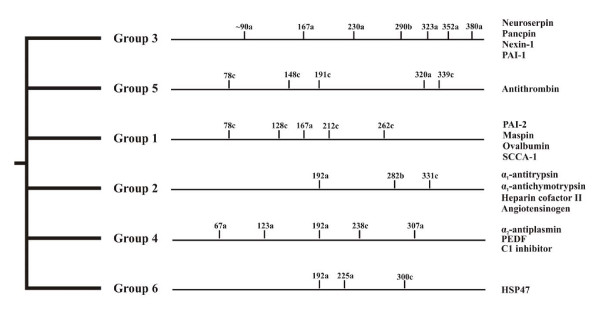
**Gene structure-based phylogenetic classification of vertebrate serpins**. Positions of introns refer to the human α_1_-antitrypsin sequence. A two amino acid indel present between positions 173 and 174 (α_1_-antitrypsin numbering) suggests that groups 1, 3, and 5 are more closely related to each other than to the other groups. Gene groups 2, 4, and 6 lack the 173/174 indel and depict an intron at position 192a, implying shared ancestry. Some group 1 members contain an additional intron at position 85c (not shown). For further details see references 20 and 21.

In metazoans, *serpin *genes display highly variant exon-intron patterns that, however, may be strongly conserved within some taxons. Gene architecture and other rare genetic characters constitute a robust basis to group vertebrate serpins [20-22]. Grounded on number, positions, and phases of introns, serpins have been classified into six groups maintained at least since the fish/tetrapod split (Figure 1). Vertebrate *serpin *genes with equivalent gene structures often tend to be organised in clusters [22-24]; however, close physical linkage is not always found. Interestingly, none of altogether 24 intron positions mapping to the core domain of vertebrate serpins is shared by all of these six gene groups; however, characteristic amino acid indels provide some further cues for unraveling phylogenetic relationships [20]. None of the group-specific vertebrate gene architectures is found in earlier diverging animal taxons, though a few vertebrate-specific intron positions are present in a scattered fashion in some basal metazoans. Another classification system groups vertebrate serpins into nine clades [1]. However, a deeper rooting, resilient phylogenetic classification of metazoan serpins is not available and their evolutionary roots are unresolved. In addition, there is no data indicating when and how the highly conserved exon-intron patterns of the paralogous vertebrate *serpin *gene groups arose. Here, data are presented that reveal a deeply rooting, continuous lineage of secretory pathway-associated serpins in metazoans that provide a surveillance and controlling function against proteolytic activity within the major cellular export/import routes.

## Results

### Chromosomal arrangement of genes coding for neuroserpin homologs along the lineages leading to vertebrates

**Figure 2:**
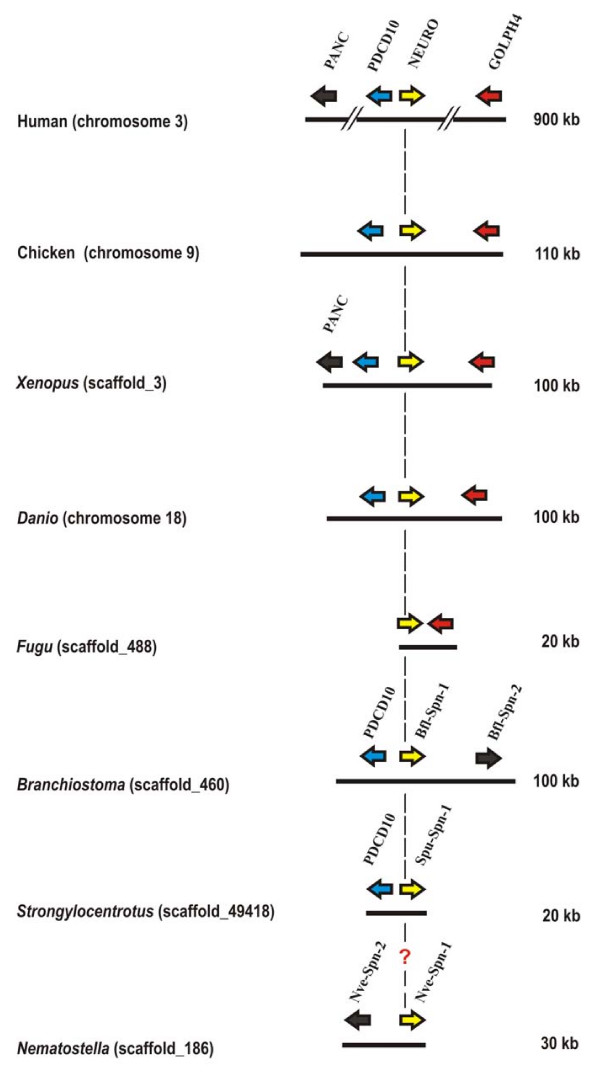
**Genomic coordinates of the genes coding for neuroserpin homologs and flanking genes in metazoans**. A vertical dashed line indicates neuroserpin (NEURO) orthologs. The genes coding for orthologs of neuroserpin and PDCD10 are consistently arranged in a head-to-head orientation at least since divergence of vertebrates and sea urchins. Orthologs are represented in identical colors. Serpin paralogs are represented as black arrows. The genes coding for neuroserpin and pancpin (PANC) share the characteristic intron distribution pattern of group 3 serpins maintained at least since the fish/tetrapod split.

Group 3 of mammalian *serpin *genes contains five members (*plasminogen activator inhibitor-1/SERPINE1*, *nexin-1/SERPINE2*, *SERPINE3*, *neuroserpin/SERPINI1*, *pancpin/MEPI/SERPINI2*) that share a highly conserved group-specific exon-intron pattern characterised by the presence of six introns at equivalent positions. Another probably homologous intron mapping to the N-terminal region cannot be positioned unambiguously due to alignment problems [20]. In the human genome, the genes coding for neuroserpin and pancpin are co-localized in opposite directions on chromosome 3 http://www.ncbi.nlm.nih.gov/projects/mapview/map_search.cgi?taxid=9606. Between these two *serpin *genes and immediately adjacent to the *neuroserpin *gene, but in inverse orientation, the *PDCD10 *(*programmed cell death 10*) gene is found (Figure 2). *PDCD10 *is a strongly conserved gene with orthologs in both vertebrates and invertebrates. In humans, the gene product has been shown to be part of a signaling complex involved in vascular development [25]; however, the exact function is unclear and paralogs are not known [26]. Mutations in the *PDCD10 *gene cause cerebral cavernous malformations (CCM), a syndrome associated with seizures and neurological deficits due to focal haemorrhages [26]. Downstream from the *neuroserpin *gene and in inverse orientation, the *GOLPH4 *marker is found that codes for a transmembrane protein (GPP130) involved in endosome-to-Golgi traffic of proteins [27-29].

**Figure 3:**
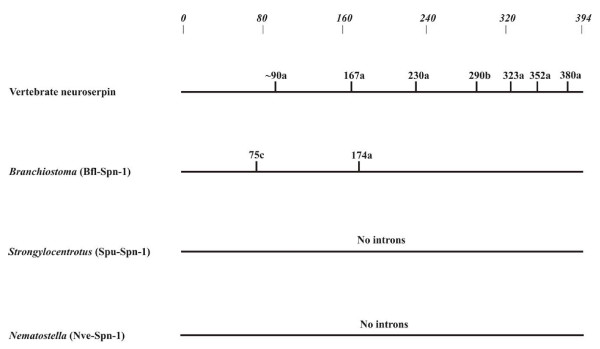
**Exon-intron organisation of the *neuroserpin *gene lineage**. The *Nematostella vectensis serpin *gene *Nve-Spn-1 *is included, though orthology with the deuterostome counterparts is currently only supported by protein-based signature sequences. Specifications for intron positions and their phasing refer to mature human α_1_-antitrypsin. Only introns mapping to the serpin core domain (residues 33 to 394 of the reference) are considered.

**Figure 4:**
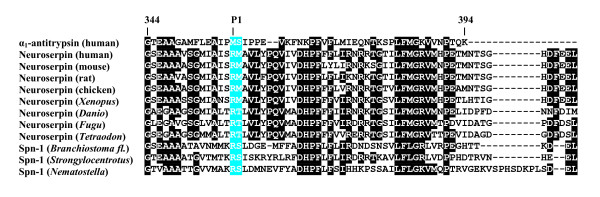
**C-terminal sequences of neuroserpin orthologs from deuterostomes and serpin Spn-1 from *Nematostella vectensis***. The numbering of amino acids refers to human α_1_-antitrypsin (top). Amino acids flanking the (putative) scissile bond are marked in turquoise, and the P1 position is indicated. Residues conserved in at least 70% of sequences are reproduced in white-on-black.

Studying the serpin complement of various metazoans we noted that linkage of the *PDCD10*-*neuroserpin*-*GOLPH4 *triad is maintained in the genomes of chicken, the clawed frog (*Xenopus tropicalis*), and the zebrafish (*Danio rerio*). Current genome sequence releases also revealed synteny of the *pancpin *gene with these three genes in the genome of the clawed frog (*Xenopus tropicalis*), and conservation of the close head-to-head association of the *neuroserpin/GOLPH4 *gene pair in the Japanese pufferfish (*Fugu rubripes*) (Figure 2) and in *Tetraodon nigroviridis*. Orthology of *neuroserpin *genes in these species is corroborated by the highly conserved group 3 specific exon-intron gene architecture (Figure 3), and a C-terminal extension (Figure 4) that targets neuroserpin to large dense core vesicles in mammals [11].

Extension of microsynteny analysis to lancelets (*Branchiostoma floridae*) and sea urchins (*Strongylocentrotus purpuratus*) showed that a *serpin *gene is present in either of these species in close vicinity to the *PDCD10 *gene (Figure 2). As in vertebrates, these genes are arranged in a head-to-head orientation. Sequence comparisons corroborated that the *Branchiostoma floridae serpin *adjacent to *PDCD10*, denominated *Bfl-Spn-1*, is the ortholog of the previously characterised *serpin *gene *Spn-1 *from the closely related *Branchiostoma lanceolatum *(92.2% sequence identity for the C-terminal 385 amino acids) that was recently shown to inhibit proprotein convertases [30]. Each of these serpins contains a highly conserved RSL region (positions P5 – P1': NMMKR ↓ S), and a C-terminal ER retention/retrieval signal (KDEL) (Figure 4). The presence of an N-terminal signal peptide in lancelet Spn-1 mediating access to the secretory pathway is supported by cDNA sequence analysis and expression studies [30]. The gene cluster harbouring the *PDCD10/Spn-1 *gene pair includes a closely related paralog (*Bfl-Spn-2*) of *B. floridae Spn-1 *(Figure 2) that also has a counterpart in *B. lanceolatum *(not shown).

**Figure 5:**
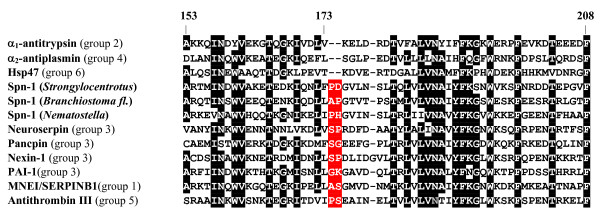
**A discriminatory indel supports relationships of neuroserpin and homologs from sea urchins, lancelets, and *Nematostella***. Human representatives of vertebrate serpin groups 1, 3, and 5 containing the indel (marked in red), and from groups 2, 4 and 6 that lack the indel, are shown. The numbering of positions shown above the alignment refers to the sequence of mature human α_1_-antitrypsin. Positions conserved in at least 70% of sequences are represented in white-on-black printing.

Similar to lancelets, the genome of the sea urchin *Strongylocentrotus purpuratus *revealed linkage of the *PDCD10 *gene to an inversely oriented *serpin *gene, named *Spu-Spn-1 *(accession number: XP_001186705) within a 20 kb DNA segment. Apart from synteny of its gene with *PDCD10*, Spu-Spn-1 shares a signal peptide, a conserved RSL region including the dibasic KR motif preceding the inhibitor's putative scissile bond (P5-P1': TMTKR ↓ S), and a variant (HEEL) of the canonical KDEL signal with the corresponding lancelet serpin (Figure 4). The HEEL motif was recently shown to mediate ER retention in transfected HeLa cells [31]. Another feature corroborates evolutionary continuity extending from mammalian neuroserpin via *Branchiostoma *Spn-1 to Spu-Spn-1 from the sea urchin. Groups 1, 3 and 5 of vertebrate serpins are discernible from groups 2, 4, and 6 by a two amino acid indel following residue 173 (α_1_-antitrypsin numbering, ref. [20]). The discriminating dipeptide sequence (previously assigned adjacent to position 171, due to use of a different set of aligned serpin sequences) is also found in *Branchiostoma *Spn-1, and in Spu-Spn-1 from *Strongylocentrotus purpuratus *(Figure 5). Collectively, these data suggest that a secretory pathway-associated serpin that already existed at least since the emergence of deuterostomes gave rise to mammalian neuroserpin.

**Figure 6:**
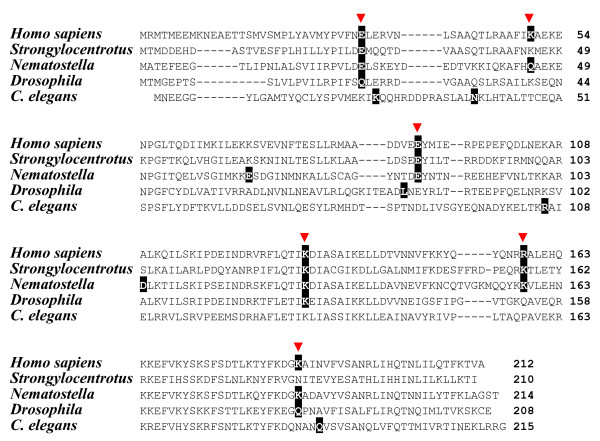
**Intron positions of *PDCD10 *genes in metazoans**. Intron positions (white-on-black printing, phasing not indicated) were identified with GENEWISE and mapped onto the protein sequences. Intron positions conserved in at least two species are marked with an arrow head. Accession numbers for PDCD10 sequences: AAH16353 (human); XP_001186662 (*Strongylocentrotus purpuratus*); EDO34838 (*Nematostella vectensis*); AAF55190 (*Drosophila melanogaster*); CAA90115 (*C. elegans*).

Inspection of genomes from deeper rooting metazoans revealed *PDCD10 *orthologs in *Drosophila melanogaster *and in *C. elegans *(Figure 6), depicting 49% and 39% sequence identity at the protein level with their human counterpart [26], but close linkage of this marker to a *serpin *gene is neither evident in the fruit fly nor in the worm. In *Drosophila melanogaster*, genes of unknown functions flank *PDCD10*, and in the nematode genome, the *PDCD10 *microenvironment differs from that of both *Drosophila *and that of vertebrates (see Additional file 1), making identification of neuroserpin orthologs in these species more difficult. Some data, however, suggest the existence of a neuroserpin ortholog at least since the divergence of sponges and eumetazoans, believed to have occurred at least 650 to 700 million years ago [32-34]. To date, three *serpin *genes have been identified in the genome of the sea anemone *Nematostella vectensis *[34], one of which (accession number: XP_001627732; http://genome.jgi-psf.org/Nemve1/Nemve1.home.html: estExt_fgenesh1_pg.C_1860016) displays features suggesting shared ancestry with neuroserpin orthologs. These features include a dibasic amino acid sequence motif preceding the putative scissile bond, and a C-terminal extension ending with the tetrapeptide sequence SDEL, a functional variant of the canonical ER retention/retrieval signal [31]. This serpin (as its sea anemone paralogs) also possesses the dipeptide indel adjacent to position 173; however, further sequence-independent data are needed to firmly establish the presumed type of kinship.

### The exon-intron structures of the adjacent *neuroserpin *and *PDCD10 *gene orthologs underwent different fates during deuterostome evolution

Though microsynteny and signature sequences strongly argue in favour of a common ancestor giving rise to mammalian neuroserpin, Spu-Spn-1 from *Strongylocentrotus purpuratus*, and Spn-1 from *Branchiostoma*, their genes depict quite different patterns of intron distribution (Figure 3). The sea urchin *Spu-Spn-1 *gene does not contain any intron mapping to the serpin core domain, and the single (correctly predicted?) intron resides in the sequence coding for the signal peptide (accession number: NW_001288761). The *Spn-1 *gene from lancelets harbours introns at positions 75c and 174a (α_1_-antitrypsin numbering). This intron-poor gene architecture contrasts with the mammalian *neuroserpin *gene that depicts the characteristic group 3 exon-intron structure with introns at positions 167a, 230a, 290b, 323a, 352a, and 380a (the first intron of the *neuroserpin *gene mapping to the serpin core domain, tentatively assigned to position ~90a, cannot be assigned reliably, due to alignment ambiguities). Strikingly, none of these intron positions is conserved among *neuroserpin *orthologs from lancelets, sea urchins or vertebrates. There is also no congruence of the introns at positions 75c and 174a in the lancelet *Spn-1 *gene with any of the other vertebrate *serpin *genes (Figure 1). Obviously, massive changes have occurred along the *neuroserpin *gene lineage concerning exon-intron organisation since divergence of echinoderms, cephalochordates and vertebrates. The *Spn-1 *gene from *Nematostella vectensis *also does not contain an intron mapping to the serpin body (Figure 3).

Contrasting with the *neuroserpin *gene lineage, comparably few changes are evident in the architecture of the immediately adjacent *PDCD10 *gene since the split of sea urchins and mammals (Figure 6). The *PDCD10 *genes from humans and *Strongylocentrotus *have four out of six intron positions in common. Two introns (positions 50c and 186b, numbering based on the human sequence) seem to have been lost in the sea urchin, since they are present in the earlier diverging cnidarian, *Nematostella vectensis*. The sea anemone *PDCD10 *gene contains eight introns, six of which are found at equivalent positions in the human homolog. *Nematostella vectensis *genes were recently demonstrated to share the majority of intron positions with their mammalian counterparts [34]. None of the *PDCD10 *introns of *C. elegans *superimposes on an intron found in the orthologs from humans, the sea urchin, or the sea anemone.

## Discussion

The findings here reveal a clear history of neuroserpin, a prominent group 3 vertebrate serpin. Features derived from the genomic, gene and protein level provide ample discriminatory data to enable drawing of a reliable kinship history of its previously unknown origin. Microsynteny analysis proved to be especially illuminating, demonstrating that rare genomic characters can provide very useful information for decoding of bonds in protein families with intricate evolutionary history. Recent investigations provide a plausible explanation for the strongly conserved syntenic association of *PDCD10 *and *neuroserpin *orthologs during diversification of deuterostomes. Apparently, expression of the head-to-head arranged genes is controlled by a bi-directional, asymmetrically acting promoter region inserted within the ~0.9 kb intergenic region separating the transcription units coding for PDCD10 and neuroserpin [35]. Dependence on the common regulatory region thus may have forced the maintenance of linkage of these genes. The rapidly increasing flood of data from genome sequencing projects will certainly continue to provide further discriminatory information from multiple, independent levels of biological organisation, such as codon usage dichotomy [36], to enable robust classification of other metazoan serpins.

Neuroserpin orthologs from early diverging deuterostomes, like *Strongylocentrotus *or *Branchiostoma*, contain classical ER retention signals (KDEL or HEEL) at their C-terminal ends, and the *Nematostella *Spn-1 sequence terminates with SDEL, which functions as an autonomous ER retention/retrieval signal in HeLa cells, when hooked to a reporter protein [31]. The C-terminal end of neuroserpin from mammals, chicken, and *Xenopus *is HDFEEL (Figure 4). In HeLa cells, which express three different KDEL receptors with overlapping, but not identical passenger specificities, the FEEL sequence targets attached passenger proteins primarily to the Golgi, though some 25% of cells depict ER localisation [31]. In transfected COS cells, intracellular neuroserpin localises to either the ER or Golgi [11]; in cells with a regulated secretory pathway, however, neuroserpin resides in large dense core vesicles, mediated by a C-terminal extension encompassing the last 13 amino acids, including the FEEL sequence [11]. Collectively, these data are compatible with the view that, in an ancient ortholog of neuroserpin, a two amino acid insertion (FE) gave rise (in combination with additional residues?) to a modified sorting signal enabling a more specialised subcellular localisation. Irrespective of the still fragmentary data concerning the phylogenetic classification of Spn-1 from the sea anemone, it is clear that surveillance of the secretory pathway routes by serpins is an ancient and conserved trait in eukaryotes. Whether the C-terminal extensions of neuroserpin orthologs from fishes (Figure 4) are functional secretory pathway address signals remains to be determined.

The regional changes of placement within the secretory route may have come along with diversifications associated with the inhibitors' functions due to changes within the RSL region. Neuroserpin from vertebrates is believed to interact with its preferred target enzyme, tPA, via the single Arg residue (P1 position) in the RSL region [9]. In lancelets, the scissile bond is preceded by the dipeptide motif Lys-Arg (KR), which is characteristic for substrates and inhibitors of proprotein convertases, which indeed, have been identified as target enzymes of lancelet Spn-1 [30]. Similar biochemical properties are expected for Spn-1 from the sea urchin, and Spn-1 from the sea anemone (Figure 4). The physiological interaction partners of these inhibitors have not yet been identified.

Though the data clearly indicate that the roots of mammalian neuroserpin may be traced back far in the history of animals, unequivocal support for a neuroserpin ortholog in arthropods is still lacking. Several labs have provided evidence for a serpin (Spn4) with furin inhibiting activity and containing a canonical ER targeting signal in *Drosophila *[13-15], and a similar protein has been detected in *Anopheles *[37]. However, caution should be advised, because homoplasy due to convergent evolution currently cannot be excluded. The *Spn4 *gene is prone to recombination events, especially in the regions coding for the RSL region [19]. Unraveling the relationships of the *Spn4 *gene from fruit flies and *neuroserpin *orthologs from deuterostomes requires further investigation.

The history of the *neuroserpin/PDCD10 *gene pair reveals some remarkable insights into the evolution of the exon-intron structure of metazoan genes. Even closely adjacent genes that are physically linked at least since divergence of echinoderms and chordates may be subject to quite different trends affecting the intron distribution patterns. Comparably few changes in the exon-intron architecture have happened in *PDCD10 *orthologs since divergence of lineages leading to sea anemones and vertebrates (Figure 6). In *PDCD10 *genes, six out of eight intron positions occurring in humans or in the cnidarian are conserved. This is in accordance with findings demonstrating that the majority of genes from early diverging present-day eumetazoans are intron-rich with most introns apparently maintained since ancient times [34,38]; for *serpin *genes, however, the situation appears to be different. Regardless of the still rudimentary evidence for the putative sea anemone *neuroserpin *ortholog, the available data show that *serpin* genes in *Nematostella vectensis *are intron-poor. The sea anemone *Spn-1 *gene does not contain any introns mapping to the serpin body, and the single serpin core intron identified in one (accession number: XP_001627750) of the currently known three *Nematostella vectensis serpin *genes maps to residue 42c (α_1_-antitrypsin numbering; not shown). Looking up at deuterostomes, the sea urchin *neuroserpin *ortholog *Spu-Spn-1 *is also devoid of introns within the region coding for the serpin core. In contrast, the *Spn-1 *genes from *Branchiostoma floridae *(Figure 3) and its close relative, *Branchiostoma lanceolatum *[30] each depict two introns mapping to identical sites within the serpin body. Their positions, however, are not congruent with any of the introns of mammalian *neuroserpin*, the prototype group 3 vertebrate *serpin *gene or with any other intron location known from vertebrate *serpin *genes [20]. Therefore it must be considered that, in the serpin lineage leading to mammalian *neuroserpin*, an appreciable fraction of introns is not ancient, but may have been acquired during metazoan evolution; however, it cannot be excluded that intron paucity in present-day *serpin *genes of cnidarians (and in *neuroserpin *orthologs from sea urchins and lancelets) is due to massive intron loss, in contrast to most other introns that have survived hundreds of millions of years in these creatures. Intron gain is possibly not as rare as sometimes believed [39], however, it could be confined to certain gene families and/or to discrete evolutionary phases [40], for as yet unexplored reasons. Several types of processes have been proposed that may explain how introns may be acquired, but definite answers are still awaited.

## Conclusion

In this study, we analysed and resolved the evolutionary roots of neuroserpin, a secretory-pathway associated mammalian serpin. Insight into the intricate history of the multi-membered serpin superfamily beyond the fish/tetrapod split was obtained by showing that orthologs of neuroserpin exist at least since the emergence of deuterostomes and probably already since divergence of eumetazoans and *Bilateria*. The continuous presence of neuroserpin orthologs equipped with C-terminal signal sequences assigning residence within the secretory pathway documents that serpins functioning as guards of the cellular export/import routes represent an ancient trait. This surveillance role has been subject to subtle functional and local variances during evolution as evidenced by changes within the RSL and the subcellular address signal. In contrast to many other, even closely linked genes, in which the majority of intron positions has been conserved for hundreds of millions of years, the intron distribution pattern of *neuroserpin *gene orthologs has experienced massive changes, perhaps dominated by intron gain.

## Methods

### Identification of serpin DNA and protein sequences and microsynteny analysis

Serpin protein and DNA sequences of various genomes were extracted from publicly accessible databases (see Additional file 2) via the BLAST software package (including PSI-BLAST) using key words or the human α_1_-antitrypsin sequence for searching. Chromosomal microsynteny analysis was performed using the NCBI Map Viewer [41], the ENSEMBL genome browser [42], the JGI genome browser [43], the *Tetraodon *genome browser [44], the UCSC genome browser [45], and inspecting the *Strongylocentrotus purpuratus *genome database [46].

### Sequence alignments, gene structure analyses and mapping of intron positions

Alignments of protein sequences were performed with CLUSTAL X [47] and refined manually in GeneDoc [48]. Intron positions were identified and assigned with GENEWISE [49]. Mature human α_1_-antitrypsin was used as reference for mapping of positions and phasing of introns in *serpin *genes [20].

## Authors' contributions

AK carried out analyses. HR conceived and supervised the project and wrote the paper. Both authors have read and approved the final manuscript.

## Supplementary Material

Additional file 1 **Genes flanking *PDCD10 *orthologs in *D. melanogaster *and *C. elegans***. Genes flanking *PDCD10 *orthologs in *D. melanogaster *and *C. elegans*. Neighbouring genes of *PDCD10* Click here for file

Additional file 2 **Sources of data for genomes investigated in this study**. Sources of data for genomes investigated in this study. Web adresses of genomes Click here for file
