# Supplementary material for: Ancestry and evolution of a secretory pathway serpin
Source: BMC Evol Biol. 2008 Sep 15;8:250. doi: 10.1186/1471-2148-8-250 (PMC2556349; doi:10.1186/1471-2148-8-250)
Supplement: Additional file 1 — Genes flanking PDCD10 orthologs in D. melanogaster and C. elegans. Genes flanking PDCD10 orthologs in D. melanogaster and C. elegans. Neighbouring genes of PDCD10 [file 1471-2148-8-250-S1.doc]

**Supplementary Table 1: Genes flanking *PDCD10* orthologs in *D. melanogaster* and *C. elegans.***

***PDCD10* (accession no) Neighbouring genes, function**

*D. melanogaster* (*CG5073*) left: *CG14868* (HOOK protein), *CG6136* (copper metabolism?)

right: *CG14867* (unknown); *CG6130* (unknown);

*CG31302* (contains Src homology 3 domain)

*C. elegans* (*C14A4.11*) left: *C14A4.10* (taf-13, transcription factor IID family)

right: *C14A4.12* (unknown)
