# Supplementary material for: Ancestry and evolution of a secretory pathway serpin
Source: BMC Evol Biol. 2008 Sep 15;8:250. doi: 10.1186/1471-2148-8-250 (PMC2556349; doi:10.1186/1471-2148-8-250)
Supplement: Additional file 2 — Sources of data for genomes investigated in this study. Sources of data for genomes investigated in this study. Web adresses of genomes [file 1471-2148-8-250-S2.doc]

**Supplementary Table 2: Sources of data for genomes investigated in this study.**

Genome Version Website

*Homo sapiens* Build 36.2 (September 2006) http://www.ncbi.nlm.nih.gov/projects/mapview/map_search.cgi?taxid=9606

*Gallus gallus*  WASHUC2.1 (May 2006) http://www.ncbi.nlm.nih.gov/projects/mapview/map_search.cgi?taxid=9031

***Xenopus tropicalis*** v.4.1 (August 2005) http://genome.jgi-psf.org/Xentr4/Xentr4.home.html

Fugu rubripes v.4.0 (October 2004) http://genome.jgi-psf.org/Takru4/Takru4.home.html

*Danio rerio* Zv6 (March 2006) http://www.ncbi.nlm.nih.gov/projects/mapview/map_search.cgi?taxid=7955

*Tetraodon nigroviridis* Assembly V7 (April 2003) http://www.genoscope.cns.fr/externe/tetranew/

*Branchiostoma floridae* V1 (March 2006) http://genome.jgi-psf.org/Brafl1/Brafl1.home.html

*Strongylocentr. purp.* Spur_2.1 assembly (Sept. 2006) http://www.hgsc.bcm.tmc.edu/projects/seaurchin/

*Nematostella vectensis* v1.0 (**July 2007)** http://genome.jgi-psf.org/Nemve1/Nemve1.home.html

*Caenorhabditis elegans* Release WS187 (Feb. 2008) http://www.wormbase.org/
